# Humans are not unique: difficult birth is common in placental mammals

**DOI:** 10.1002/brv.70174

**Published:** 2026-05-06

**Authors:** Nicole D. S. Grunstra

**Affiliations:** ^1^ Department of Evolutionary Biology University of Vienna Djerassiplatz 1 Vienna 1030 Austria; ^2^ Mammal Collection Natural History Museum Vienna Burgring 7 Vienna 1010 Austria; ^3^ Human Evolution and Archaeological Sciences (HEAS) University of Vienna Djerassiplatz 1 Vienna 1030 Austria

**Keywords:** childbirth, dystocia, obstructed labour, fetopelvic disproportion, mammals, obstetrical dilemma, life‐history trade‐offs, neonatal size, pelvis, cliff‐edge selection

## Abstract

Human childbirth is widely presumed to be uniquely difficult and dangerous compared to birth in other mammals. Tight fetopelvic proportions can result in obstructed labour and contribute to high rates of maternal and neonatal mortality. Ideas summarised under the ‘obstetrical dilemma’ have contributed to this assumption by explaining difficult childbirth as the outcome of a unique evolutionary history of bipedalism and encephalisation. However, a comprehensive review of the literature and an analysis of collated data demonstrate that human birth is not truly exceptional in its risk. Dystocia (birth difficulties) occurs across a phylogenetically wide range of placental mammals, including wild populations where natural selection would be expected to minimise its occurrence. Various species show similar or higher dystocia prevalence compared to humans, and fetopelvic disproportion is particularly common in species with precocial, large‐bodied offspring. The underlying causes and risk factors are also highly comparable. Contrary to long‐standing assumptions, natural selection has not eliminated dystocia in natural mammal populations, suggesting that parturition in placentals may be characterised by a persistent baseline risk of complications. This persistent risk likely reflects a life history trade‐off between the improved survival of large offspring and the occasional costs of birth complications. A cliff‐edge model of selection can explain the evolutionary dynamics underlying this trade‐off and the persistent fraction of fetopelvic disproportion and related forms of obstructive dystocia. An additional trade‐off involving litter size is evident in litter‐bearing, often altricial species, where both small and large litters increase the risk of dystocia. Phenotypic plasticity further maintains dystocia in populations despite selection, while also contributing to variation in birth difficulty within and among species. These findings reposition human birth difficulties within a broader mammalian pattern rather than as a uniquely human phenomenon requiring exceptional explanations. Reframing human childbirth in this broader context challenges entrenched assumptions and highlights the value of a comparative evolutionary perspective.

## I. INTRODUCTION

Difficult childbirth is considered a hallmark of human reproduction. Birth in humans is relatively risk‐prone and involves multiple potential complications and associated medical resolutions. Every year, mothers and babies die or sustain long‐term injuries due to childbirth. Maternal mortality – maternal death due to pregnancy or childbirth – remains high globally. In 2000, the maternal mortality ratio was nearly 340 per 100,000 live births, while in 2020, an estimated 223 mothers per 100,000 live births still died, or about 786 women per day (World Health Organization, 2023). Birth‐related perinatal mortality of the fetus or neonate is even higher (Hug *et al*., 2021; UNICEF, 2025).

There is a widespread notion that birth in humans is uniquely difficult, in degree or kind, compared to our mammalian relatives. In addition to an anthropocentric world view, this stems from the long‐standing and influential idea of an evolutionary trade‐off summarised under the ‘obstetrical dilemma’: the human pelvis evolved its characteristic shape to accommodate bipedal locomotion, while our encephalisation entails giving birth to large‐brained fetuses. As a result, human babies have big heads relative to the dimensions of the maternal birth canal. Indeed, it was long thought that human fetuses are born ‘early’ and in a helpless state compared to other primate neonates to avoid outgrowing the maternal pelvis (Washburn, 1960). Complicated and ‘risky’ childbirth is thus assumed to be a product of our unique evolutionary history (Krogman, 1951; Washburn, 1960; Rosenberg, 1992). Across human populations, a persistent (if variable) proportion of births involves obstructed labour, which can result from fetopelvic disproportion – a condition where the fetus, typically its head, is too large to pass through the birth canal (Dolea & Abouzahr, 2003). True fetopelvic disproportion is usually fatal to both mother and child unless resolved through surgical intervention.

Difficult childbirth, or ‘labour dystocia’, refers to slow labour progress or labour complications that require assistance (Mee, 2008; Noakes, Parkinson & England, 2009; Neal *et al*., 2015; Kissler & Hurt, 2023). Dystocia can arise from several underlying physiological and anatomical causes (Zhu *et al*., 2006; Noakes *et al*., 2009). It increases the likelihood of fetal asphyxia, injury, difficulty suckling and putting on weight, as well as perinatal death (e.g. Lanci *et al*., 2022; Allen Wild & Yon, 2024). For the mother, dystocia is associated with a higher probability of injury and infection, future infertility, and death (Allen Wild & Yon, 2024). Because of these potentially devastating effects on reproductive fitness, recurring dystocia is assumed to be evolutionarily untenable and thus rare or absent in wild mammals, making humans seem like an exception (Roy, 2003; Mee, 2008).

Despite intense debate around the obstetrical dilemma, the dominant view today is that our tight fetopelvic fit emerged as an evolutionary compromise between a multitude of opposing selection pressures. Our unique bipedal posture and possibly locomotion favour a smaller lower pelvis (including the birth canal), whereas human encephalisation and high birth weight favour a more spacious birth canal (Rosenberg & Trevathan, 1996; Wells, DeSilva & Stock, 2012; Haeusler *et al*., 2021; Grunstra *et al*., 2023). However, the relevance of these evolutionary trade‐offs for understanding present‐day maternal mortality and morbidity is controversial. Aspects of our modern environments and lifestyles such as obesity, carbohydrate‐rich diets, and stunted maternal growth due to childhood malnutrition increase the present‐day risk and incidence of fetopelvic disproportion and obstructed labour (Wells *et al*., 2012; Wells, Wibaek & Poullas, 2018). Clinical management of birth itself can also complicate deliveries (Dunsworth, 2016; Stone, 2016; Walrath, 2017). Nonetheless, these ecological and cultural factors do little to challenge the belief that parturition in humans is more difficult and risk‐prone compared to that in other mammals. The fact that only humans routinely engage in birth assistance (midwifery) across cultures reinforces this perception (Rosenberg & Trevathan, 1996; Trevathan, 2011). Moreover, medical interventions such as caesarean sections have partially relaxed natural selection on birth canal and fetal dimensions, increasing the expected frequency of fetopelvic disproportion over time (Mitteroecker *et al*., 2016).

**Fig. 1:**
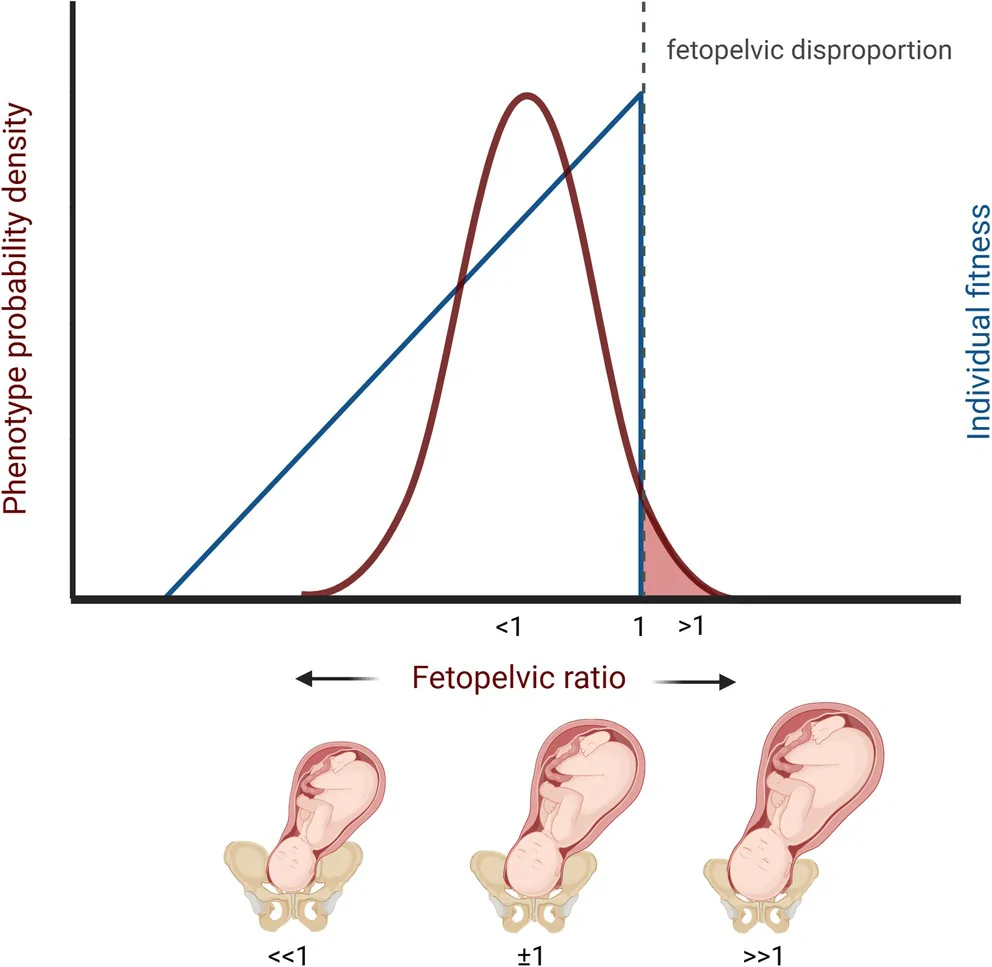
Cliff‐edge model of selection. Individual fitness increases with newborn size and with a smaller maternal pelvis until offspring dimensions exceed those of the maternal birth canal. Once fetopelvic disproportion occurs, fitness drops sharply. Population fitness is maximised when the highest frequency of individuals has the largest possible neonate that can still pass through the birth canal. However, because fetal and pelvic dimensions vary continuously in the population, a small proportion of individuals will experience fetopelvic disproportion (red shaded area on graph). The fetopelvic fit is illustrated with three examples. Variation in the relationship between fetal size and birth canal dimensions can arise from differences in fetal size, maternal pelvic canal size, or both. Created in https://BioRender.com, after Mitteroecker *et al*. (2016).

The persistence of obstructed labour despite its fitness costs has been modelled by the concept of cliff‐edge selection (Mitteroecker *et al*., 2016), which provides a useful framework for understanding how such difficulties might arise and persist in humans and potentially other mammals. Increased birth weight confers improved health and survival chances in humans (Barker *et al*., 1989; Alberman, 1991). Human pelvic canal dimensions are likely constrained by the need for pelvic floor support, possibly other aspects of bipedalism, as well as thermoregulation (reviewed in Wells *et al*., 2012; Betti, 2017; Haeusler *et al*., 2021). Under cliff‐edge dynamics, evolutionary fitness rises with increasing neonatal size until it reaches a threshold imposed by maternal pelvic dimensions, beyond which obstructed labour occurs and fitness drops sharply, producing an asymmetric fitness curve (Fig. 1). While the highest individual fitness is thus achieved with a maximally tight fetopelvic fit that still allows successful birth, evolution favours trait combinations that maximise mean fitness in the population. This involves fitting the normally distributed phenotypes of fetal and pelvic size to the asymmetric fitness curve. As a result, even when average phenotypes are near the fitness optimum (the ‘cliff edge’), a small but persistent proportion of individuals will experience fetopelvic disproportion and birth‐related mortality, i.e. those who ‘fall off the fitness cliff’ (Mitteroecker *et al*., 2016; Fig. 1). This scenario offers a compelling explanation for the continued presence of obstructed labour, even in the absence of routine medical intervention.

Nevertheless, whether human childbirth is exceptionally prone to complications – and whether this stems from unique evolutionary or biocultural factors – remains untested. The related assumption that natural selection eliminates birth‐related mortality in wild mammals also lacks empirical support and was recently challenged (Grunstra, 2024; Laudicina *et al*., 2025). If the cliff‐edge model captures a general evolutionary dynamic, then humans may not be the only species vulnerable to persistent fetopelvic disproportion despite selection against it. If this difficulty was primarily a consequence of bipedalism or extreme encephalisation, we would expect fetopelvic disproportion to be rare in non‐bipedal, small‐brained taxa. Many non‐primate placental mammals, including ungulates, seals, and especially bats, give birth to large neonates relative to maternal size, suggesting they too may face biomechanical constraints and risk of obstructed labour, irrespective of brain size (Grunstra *et al*., 2019). Cliff‐edge dynamics and detectable rates of dystocia likely apply in these cases as well – including in the wild – where selection favours large neonates but pelvic canal dimensions are constrained by other functional demands – even if these do not relate to bipedalism in non‐human mammals. These scenarios remain under‐investigated but are the focus of the present review.

Attempts have been made to investigate whether other primates with relatively large brains have similar birth difficulties (Aksel & Abee, 1983; Stoller, 1996). Primate births are difficult to observe in the wild, but obstructed labour and maternal mortality have been documented in captivity, notably in squirrel monkeys (*Saimiri boliviensis* and *S. sciureus*) and macaques (e.g. *Macaca nemestrina*) (Aksel & Abee, 1983; Stockinger *et al*., 2011). However, in non‐human primates, dystocia appears more commonly due to fetal malpresentation, e.g. breech position, than fetopelvic disproportion (Stockinger *et al*., 2011; Schlabritz‐Loutsevitch *et al*., 2018). These studies are limited to a few species in artificial environments, so definitive conclusions about the risk of adverse birth outcomes in humans compared to other primates, let alone mammals more broadly, cannot be drawn.

Recent anthropological research has shifted to a more detailed study of birth canal shape and fetopelvic ratios in non‐human primates and fossil hominins, as a measure of the potential for obstructed labour (Wells *et al*., 2012; Zollikofer, Scherrer & Ponce de León, 2017; Laudicina, Rodriguez & DeSilva, 2019; Frémondière *et al*., 2022; Laudicina & Cartmill, 2023; Webb *et al*., 2024; Laudicina *et al*., 2025). While fetopelvic ratios are relatively easy to determine in a wide range of species and indeed vary among primates, associated data on birth‐related mortality and morbidity remain largely lacking. It is thus unclear how well these ratios function as a proxy for obstructed labour. For example, one recent study reported that a semi‐free‐ranging population of 112 female Japanese macaques (*Macaca fuscata*), a primate also characterised by a tight fetopelvic fit, had zero maternal mortality over a 27‐year period (Pink *et al*., 2024). Comparisons of parturition‐related complications and mortality risk between humans and other mammals are thus best grounded in direct observations of birth outcomes rather than inferences from morphological proxies alone.

Through a comprehensive review of existing literature and quantitative data, I provide an evolutionary context for dystocia, including underlying causes and risk factors across mammals. Dystocia is not unique to humans but is phylogenetically widespread among placental mammals. Fetopelvic disproportion observed in wild mammals refutes the assumption that the former is eliminated by natural selection. Lastly, many risk factors of dystocia are common to humans and other placentals, implying shared physiology and constraints. These findings dismantle the idea that human birth is uniquely perilous. Instead, birth is a risk‐prone process in many placental mammals, likely shaped by fundamental reproductive trade‐offs. These new perspectives have important implications for anthropology, the obstetrical dilemma, and mammalian life‐history theory.

## II. METHODS

Through a comprehensive literature review and analysis of quantitative and qualitative data, I provide an evolutionary and phylogenetic context for dystocia. To compare mechanistic origins of dystocia across taxa, I review underlying causes and risk factors among humans and other placental mammals.

### (1) Definition and classification of dystocia

In both humans and non‐human mammals, dystocia is defined as prolonged or difficult labour, or the inability to expel the fetus without assistance (e.g. Neal *et al*., 2015; Mee, 2008). Dystocia encompasses a range of different complications of maternal or fetal origin (Zhu *et al*., 2006; Noakes *et al*., 2009; Neal *et al*., 2015). Functional dystocia refers to the failure of expulsive forces and arises predominantly from irregular or insufficient uterine contractions (uterine inertia). This is often associated with insufficient dilation of the cervix owing to reduced fetal‐head‐to‐cervix pressure (sometimes called cervical dystocia). Obstructive dystocia – ‘obstructed labour’ in humans – occurs when the fetus' passage through the birth canal is obstructed, most commonly due to fetal maldisposition or fetopelvic disproportion, although maternal factors like a deformed pelvis or uterine and vaginal prolapse can also cause obstruction (Jackson, 2004; Noakes *et al*., 2009; World Health Organization, 2012). Fetal maldisposition is a term most used in veterinary medicine but is widely applicable, referring to any atypical presentation, position, or posture of the fetus. In humans, anything other than vertex presentation with occiput anterior position of the fetal head is usually considered a maldisposition (Neal *et al*., 2015).

Dystocia can thus have physiological or mechanical origins, although functional dystocia can be secondary to obstructive dystocia (Noakes *et al*., 2009). Since documentation in non‐human mammals often lacks specificity about dystocia type and underlying causes, I use ‘dystocia’ to refer to birth complications in general without specifying type or cause. Where possible however, I focus on obstructive dystocia and fetopelvic disproportion due to their relevance to the human obstetrical dilemma as well as mammalian life‐history trade‐offs in birth size and cliff‐edge dynamics.

### (2) Data collection

A comprehensive literature review was conducted to compile data on dystocia in humans and across as many live‐bearing mammalian taxa as possible. Data sources included veterinary and animal husbandry literature, zoological medicine reports, wildlife biology studies, and human obstetric literature. In addition to reports by recognised human public health and epidemiology authorities (e.g. the World Health Organization, the Global Burden of Disease initiative) and veterinary textbooks, the scientific literature was examined for relevant data. Search terms included ‘dystocia’, ‘obstructive dystocia’, ‘fetal maldisposition’, ‘fetopelvic disproportion’, ‘parturition’, ‘birth’, ‘mortality causes’, and ‘stillbirth’ in combination with names of species or mammalian groups. Human‐specific terms like ‘obstructed labour’ (which automatically returned results for both ‘labour’ and ‘labor’) and ‘cephalopelvic disproportion’ were also used. As stillbirth in mammals – including in humans – is considered a possible outcome of dystocia as well as parturition‐unrelated causes (Kock *et al*., 1988; Sesbuppha *et al*., 2008; Lawn *et al*., 2016; Houck & Harrison, 2021), care was taken to differentiate between these, where possible. One way was to find descriptions of birth complications such as observed obstructions. Another was to look for specific clinical signs in necropsy reports, including meconium aspiration in the lungs, oedema, bruising, haemorrhage, and fetal oversize, among others. These are well‐established indicators of intrapartum or perinatal death associated with dystocia, especially when observed in the absence of other findings [e.g. infection, pathogens, congenital anomalies (Stockinger *et al*., 2011; Rideout, 2012; Williams, 2012)].

To gain the most complete possible picture of the prevalence of dystocia and its impact on survival and reproduction across mammals, information on several different parameters of prevalence and mortality rates was collected. These variables aimed to measure the complications and risks associated with parturition and could be compared across species (Table 1). Data were either taken directly from reports in the literature or were calculated based on raw data provided in the latter. Finally, proximate underlying causes (e.g. uterine inertia, fetopelvic disproportion) and shared risk factors of dystocia (e.g. primiparity, maternal overweight) between humans and other mammals were reviewed.

**Table 1:** Definition of variables.

Variable	Definition
Dystocia prevalence	Percentage of all births that were dystocic, not including pregnancy complications [e.g. (pre)eclampsia, placenta previa]
Obstructive dystocia prevalence	Percentage of all births that were marked by obstructive dystocia
Fetopelvic disproportion prevalence	Percentage of all births that were marked by fetopelvic disproportion
Maternal mortality	Percentage of all births resulting in maternal death associated with parturition^a^
Dystocia‐related adult female mortality	Percentage of all adult (i.e. of reproductive age) female deaths attributable to dystocia^b^

^a^ For humans, the definition of the International Classification of Diseases is used: maternal deaths directly or indirectly caused by birth as well as pregnancy (World Health Organization, 2023). Dystocia is an important but not the only contributor to maternal mortality in humans.

^b^ In humans, dystocia‐related adult female mortality was defined as female deaths between 15 and 49 years of age (Global Burden of Disease Collaborative Network, 2022).

Data were collected for five categories of populations: (*i*) humans; (*ii*) domesticates (e.g. cattle *Bos taurus*, sheep *Ovis aries*); (*iii*) farmed mammals (e.g. red deer *Cervus elaphus*, or capybara *Hydrochoerus hydrochaeris*); (*iv*) captive/zoo wildlife (including primate colonies in research laboratories); and (*v*) wild/free‐ranging mammals. Statistics derived from wild populations form a crucial reference point for natural and evolutionarily relevant conditions. Conversely, domesticates are subject to artificial selection and their reproductive outcomes are therefore not fully representative of their wild progenitors. They are included here because ample and detailed data are available for them, and because of several relevant parallels to many modern human populations. Firstly, they may share ecological conditions such as the absence of predation, reduced physical exercise, increased energy intake, and routine medical intervention. Secondly, obstetric assistance and intervention are routine in many domestic populations. A comparison of the frequencies, proximate causes, and risk factors of dystocia between present‐day humans and domesticated species is therefore relevant and insightful.

To enable meaningful cross‐species comparisons, the metrics (e.g. prevalence, mortality rates) were standardised as much as possible despite variation in definitions and diagnostic criteria for dystocia. Where metrics were not directly comparable across species, I selected or transformed data to reflect similar ecological and clinical contexts. In cases where data were ecologically incomparable or potentially misleading (e.g. dystocia rates in a veterinary clinic patient population), such data were excluded in favour of more representative sources.

Human data are especially context specific and heterogeneous. In general, this problem was mitigated by including human data from different geographical regions covering all human populations, rather than using global averages. These data were preferentially extracted from a single authoritative source (e.g. Global Burden of Disease Collaborative Network), minimising methodological variation in collection of the original data.

Collecting data on dystocia prevalence rates in humans nevertheless posed a challenge. Firstly, no universal diagnostic criteria for dystocia exist in humans, with ‘slow labour progress’, ‘obstructed labour’, and ‘cephalopelvic disproportion’ sometimes used interchangeably (Dumont *et al*., 2001; Zhu *et al*., 2006; Neal *et al*., 2015). Another problem is that the frequent diagnosis of slow and obstructed labour in industrialised countries is biased by a desire to intervene medically (Dolea & Abouzahr, 2003; Rutherford, Asiodu & Liese, 2019), inflating dystocia and caesarean section (c‐section) rates in many parts of the world. C‐section rates also correlate with wealth and are increasingly performed for financial or litigation reasons rather than medical necessity, making them unreliable proxies for true dystocia in high‐income settings (Boerma *et al*., 2018). Therefore, statistics from regions and settings where records of dystocia and/or caesarean rates more reliably reflect true birth complications were used to represent dystocia prevalence in humans. These included, for example, sub‐Saharan Africa (Prual *et al*., 2000; Betran *et al*., 2021; Guo *et al*., 2025), mid‐20th‐century UK (Francome & Savage, 1993), and Sweden, a country with a national birth registry – thus including data that are representative of the entire population – and with comparatively modest obstetric intervention rates (Algovik *et al*., 2004). Furthermore, where available, birth‐related mortality data from hunter–gatherer populations were included, which provide relevant insight into the frequency of (fatal) birth complications in the absence of medical intervention (Early & Headland, 1998; Blurton Jones, Hawkes & O'Connell, 2002; Hill, Hurtado & Walker, 2007).

Mortality data also posed challenges. Firstly, human maternal and perinatal mortality rates are biased downwards in settings where midwifery and medical interventions are available, just as mortality rates in domesticated species are reduced through human obstetric assistance and intervention. By contrast, human mortality rates can be high in low‐resource settings due to poor nutrition, pelvic deformities (notably a contracted pelvis), as well as early marriage and first birth in young girls, among other factors. These ecological discrepancies among populations and species cannot be resolved and were mitigated here by including human maternal mortality data from all main geographical regions. Secondly, the WHO defines maternal mortality as maternal deaths related to pregnancy and birth, up to 42 days postpartum (World Health Organization, 2023). It thus encompasses maternal deaths due to a range of complications beyond dystocia, resulting in an overestimate of maternal mortality compared to non‐human data (Table 1), all else being equal. This definition was nevertheless followed here for humans because it yielded ample and reliable data.

The influence of modern nutritional factors in human societies that were likely rare – or at least less common – in our evolutionary past, notably obesity, gestational diabetes, a contracted pelvis, and extreme and rapid nutritional transitions (Wells *et al*., 2018, 2021) was intentionally minimised in the statistics where possible. Specifically, studies primarily sampling populations subject to these atypical nutritional and developmental conditions were excluded where feasible, as they are not representative of the broader human condition. While these examples of phenotypic plasticity are relevant from a clinical perspective and can interact with evolved biology (Wells *et al*., 2012; Wells, Desoye & Leon, 2024), they were excluded to maximise comparability across taxa in the quantitative analysis. However, since modern nutritional factors are important for understanding variation in childbirth complications in present‐day humans, they are included in the qualitative and comparative review of dystocia risk factors. Known cases of dystocia that resulted from pelvic deformities in non‐human mammals were also excluded from the quantitative analysis.

In veterinary medicine, diagnostic criteria also vary. For example, cattle dystocia is often diagnosed at a low threshold based on light, non‐instrumental assistance (Mee, 2008; Noakes *et al*., 2009). Therefore, only rates of severe dystocia requiring instrumental and/or veterinary assistance were included here, accepting that this may underestimate the true prevalence of dystocia.

In general, the less humans manage and observe mammalian parturition – varying from domestic to captive to free‐ranging populations – the more likely that only severe and fatal instances of dystocia are observed and reported, yielding conservative rates of dystocia and associated mortality. Indeed, diagnosed cases of dystocia in wild and semi‐free‐ranging populations are widely accepted to represent underestimates (Glickman *et al*., 1993; Audigé, Wilson & Morris, 2001a; Hoffman & Maestripieri, 2012; Huggins *et al*., 2013; Barros, 2016).

### (3) Comparative analysis

An absolute standardisation of definitions and diagnostic criteria across mammals was not possible. Acknowledging the aforementioned limitations, the present approach nonetheless allowed for meaningful qualitative comparisons between humans and other mammals, particularly in evaluating whether humans represent an outlier in terms of birth complications. The quantitative data do not serve to rank taxa by dystocia prevalence or mortality, but rather are intended as a framework for broader evolutionary and mechanistic interpretations.

Mammals are often classified as altricial or precocial according to their state of development at birth, based on a mix of traits such as gestation length, body size, sensory and motor functionality, and thermoregulation. Mammals vary along an altricial–precocial spectrum and can present a mosaic of these traits. Human newborns are frequently described as ‘secondarily altricial’ compared to other primates based on their helplessness and relatively small proportion of adult brain size achieved at birth (Portmann, 1941; Rosenberg, 1992; Haeusler *et al*., 2021; Gómez‐Robles *et al*., 2024). However, humans have a long gestation length, more or less functional sensory organs at birth, and a relatively large neonatal body size (Dunsworth *et al*., 2012; Tague, 2016; Grunstra *et al*., 2019; Rosenberg, 2021). Pigs have been described as ‘nominally precocial’, in that they are furred and capable of independent locomotion at birth, yet neonates have poor thermoregulatory capacity (Young *et al*., 2023). Swine, particularly domestic pigs (*Sus scrofa domesticus*), typically have large litters of individually small neonates (<1.8% of maternal body mass; Tague, 2016). For the present interpretation of dystocia prevalence and risk, obstetrically relevant life‐history traits are emphasised, particularly neonatal body size relative to maternal size and, to a lesser extent, litter size. From this perspective, humans are more similar to other precocial primates (Dunsworth, 2016; Rosenberg, 2021), with a tight fetopelvic fit despite a small proportion of adult brain size at birth, while pigs are more similar to altricial species than to other cetartiodactyls. Hence, the conventional classifications of secondary altriciality and nominal precociality for humans and pigs are not followed here, although it is acknowledged that humans are likely the most helpless and altricial among primates and pigs are more precocial than most altricial placentals.

To examine the evolutionary distribution of dystocia across the main mammalian lineages (orders), documented dystocia was mapped onto a current mammalian phylogeny based on molecular data (Upham, Esselstyn & Jetz, 2019). The specific taxa for which documentation of dystocia was found, including sources, are presented in the online Supporting Information Data S1 (‘Phylogenetic coverage’ sheet). Next, the prevalence of dystocia, obstructive dystocia, fetopelvic disproportion, as well as maternal and dystocia‐related female mortality rates by species were plotted. For each species, data from different studies, populations and/or years are presented separately wherever possible, to represent intraspecific variation. All data used and presented herein are included in Data S1. Phylogenetic relationships among the main clades (orders) are also provided to visualise phylogenetic patterns. Note that the exact phylogenetic position of certain clades, notably Perissodactyla (equids, rhinos and tapirs) among Laurasiatheria and Scandentia (treeshrews) among Euarchontoglires remains unresolved (Zachos, 2020).

## III. RESULTS

### (1) Phylogenetic distribution of dystocia

**Fig. 2:**
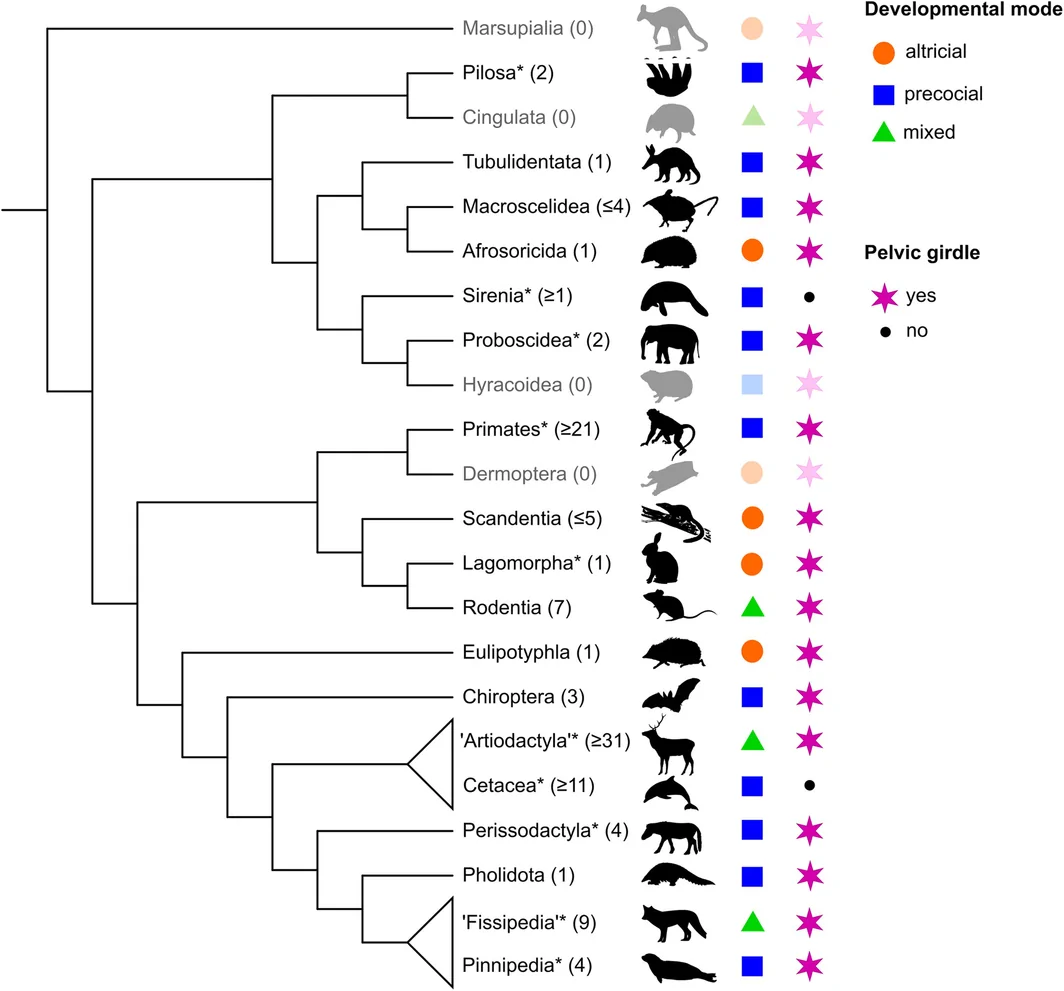
Distribution of dystocia on a phylogeny of live‐bearing mammals (marsupials and placentals). Egg‐laying monotremes are not included. Dystocia has been documented in 16 (black silhouettes) out of 19 placental orders (grey silhouettes: no record of dystocia). Numbers in parentheses refer to the number of species in which documented dystocia was found. The eight orders that include evidence of dystocia in the wild are marked with an asterisk (*). Note that the recognised orders Cetartiodactyla and Carnivora are split here into the paraphyletic ‘Artiodactyla’ (terrestrial) and Cetacea (aquatic) and paraphyletic ‘Fissipedia’ (terrestrial) and Pinnipedia (aquatic), respectively, because of strong ecological and anatomical differences. Developmental mode of newborn offspring pertains to species for which data were found (except in Marsupialia, Cingulata, Dermoptera and Hyracoidea, where developmental mode of the whole clade is represented). Dystocia even occurs in the strictly aquatic Sirenia and Cetacea, which lack bony pelvic girdles. Phylogeny from VertLife.org (Upham *et al*., 2019); branch lengths carry no information. Silhouettes are from PhyloPic.org (CC0 1.0 public domain license) and are not to scale.

Dystocia has been documented in 16 out of the 19 recognised placental mammalian orders, with dystocia in the wild observed in eight orders (Fig. 2 and Data S1). This widespread phylogenetic distribution spans terrestrial, aquatic, and flying mammals, suggesting that difficult birth is a persistent and possibly ancient feature of placental mammal reproduction, largely independent of the mode of locomotion. Dystocia predominantly occurs in precocial mammals, which give birth to a single (or few) large and well‐developed offspring (e.g. primates, elephants and cetartiodactyls, including whales), but dystocia has also been documented in altricial species, which give birth to litters of small offspring (e.g. some rodents, pigs, terrestrial carnivorans, and tenrecs *Echinops telfairi*).

Interestingly, multiple accounts of dystocia (including in the wild) are known from the two fully aquatic clades, sea cows (Sirenia) and whales (Cetacea), which lack a fully formed bony pelvis (Lacave, 1991; Hall *et al*., 2012; Sironi *et al*., 2019; Weisbrod *et al*., 2021). No evidence of dystocia exists in marsupials and indeed, dystocia is very unlikely in marsupials due to the minute size of their neonates (Burgess & Bishop, 2012; Johnson‐Delaney & Lennox, 2017). Conversely, the three placental lineages in which no dystocia was found [hyraxes (Hyracoidea), colugos (Dermoptera), and armadillos (Cingulata); Fig. 2] likely represent false negatives due to data scarcity.

### (2) Prevalence of dystocia and associated mortality in placental mammals

#### (a) Dystocia prevalence

**Fig. 3:**
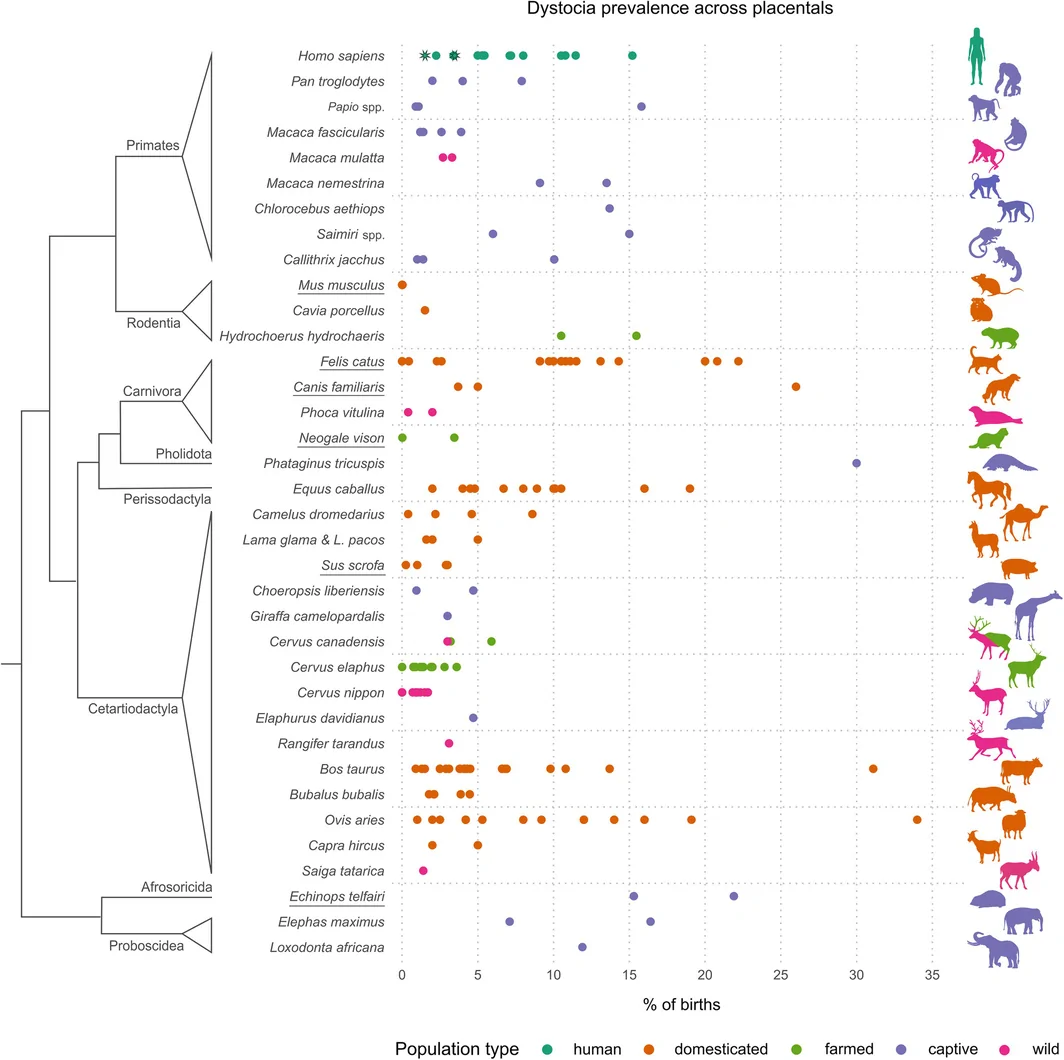
Dystocia prevalence in humans and other placental mammals. Altricial taxa are underlined. Each dot represents a study, population, or study period. Stars represent estimates from two hunter–gatherer populations living in non‐medicalised settings. High prevalence values (especially >15%) among non‐human placentals reflect specific domestic breeds or captive colonies and are unlikely to be representative of the entire species. Mammal orders are given in the cladogram (branches not scaled) and silhouettes are from PhyloPic.org (public domain) or by the author (not to scale).

Dystocia is not only phylogenetically widespread but also occurs frequently in many mammals and shows considerable overlap in prevalence between humans and other species (Fig. 3). Dystocia is common in domesticated livestock, such as cattle and sheep, and in pets. Despite their altriciality, brachycephalic (short‐faced) dog (*Canis familiaris*) and cat (*Felis catus*) breeds, such as the French bulldog or Persian cats, are well‐known to have high dystocia and c‐section rates owing to their relatively broad skulls (Noakes *et al*., 2009; Černá *et al*., 2024). Small litter size with individually large pups or kittens is another common risk factor (Münnich & Küchenmeister, 2009; Černá *et al*., 2024), although lower rates of dystocia occur across all breeds and litter sizes (Fig. 3). Captive wildlife populations, including primates and elephants, can reach dystocia rates of 15% or higher. Although less common in wild populations, serious birth complications nonetheless occur in up to 3% of births – and likely more frequently – depending on the species.

#### (b) Obstructive dystocia prevalence

**Fig. 4:**
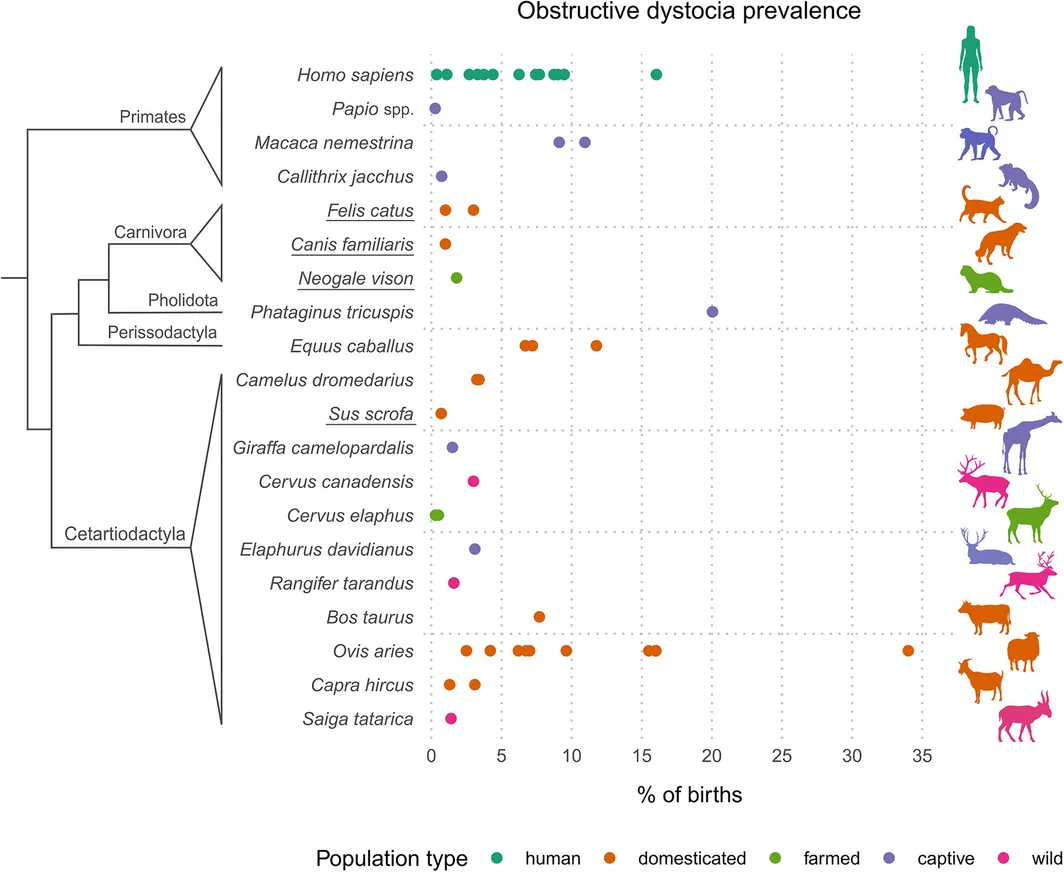
Obstructive dystocia prevalence in humans and other placental mammals. Altricial taxa are underlined. Each dot represents a study, population, or study period. Some values in the upper range for humans (*Homo sapiens*) may be overestimates as they represent geographical regions that also include upper‐middle income settings and predominantly derive from epidemiological records that may be biased upwards. High prevalence values (especially >15%) among non‐human placentals reflect specific domestic breeds, captive colonies, or small samples and are unlikely to be representative of the entire species. Mammal orders are given in the cladogram (branches not scaled) and silhouettes are from PhyloPic.org (public domain) or by the author (not to scale).

Humans and other precocial mammals, notably deer and other ungulates, experience higher rates of obstructive dystocia compared to altricial species such as dogs and pigs (Fig. 4). Although obstructed labour is diagnosed in up to 15% of human births in some regions, human variation in obstructive dystocia rates encompasses nearly the entire range observed across non‐human mammals (Fig. 4).

#### (c) Maternal mortality

**Fig. 5:**
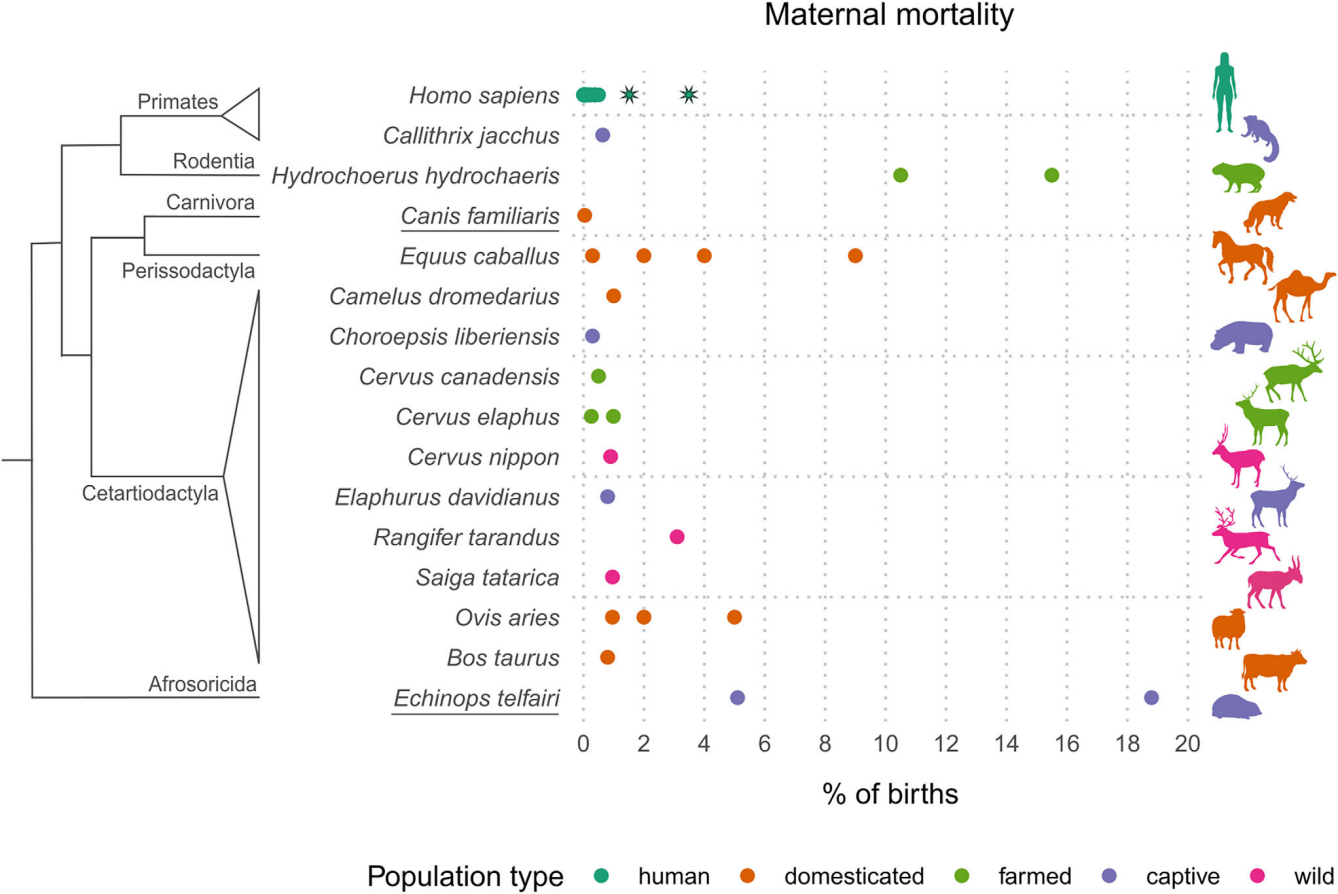
Maternal mortality in humans and other placental mammals. Altricial taxa are underlined. Each dot represents a study, population, or study period. Values represent dystocia‐related maternal mortality, except in humans (*Homo sapiens*) and horses (*Equus caballus*). Human maternal mortality includes deaths due to dystocia as well as all other deaths related to pregnancy and birth; dots represent the 21 Global Burden of Disease regions and stars represent two hunter–gatherer populations living in non‐medicalised settings. The highest values (>8%) among non‐human placentals are likely not representative of the entire species. Women and other female placentals who received life‐saving interventions are not included in the statistics presented here. Maternal mortality in the wild sika deer (*Cervus nippon*) is very likely an underestimate. Mammal orders are given in the cladogram (branches not scaled) and silhouettes are from PhyloPic.org (public domain) or by the author (not to scale).

Maternal mortality also shows considerable overlap, with several taxa showing higher values than humans (Fig. 5). However, a cross‐species comparison of mortality rates faces challenges in interpretation, principally because human data underestimate the potential for maternal mortality due to widespread obstetric intervention. This is supported by substantially higher local maternal mortality in low‐resource communities (Kassebaum *et al*., 2016; Tolentino *et al*., 2019; Wells *et al*., 2021). Nonetheless, parturition results in maternal death in non‐human mammals at meaningful frequencies, even in domesticated and farmed mammals where life‐saving intervention is also available (Fig. 5). The high rates documented in some non‐human populations are likely a consequence of their particular nutritional and captive conditions, which highlights the impact of environmental factors on birth outcomes, similar to humans (see also Section III.3 below).

Parturition in mammals is even more dangerous for the fetus/neonate (results not shown). For example, perinatal mortality – death of the offspring around the time of birth – in wild saiga antelope (*Saiga tatarica*) is at least twice as high as maternal mortality (Barros, 2016). In humans, nearly 1 per 1000 births resulted in maternal death due to obstructed labour in 2015, whereas the intrapartum stillbirth rate was ten times as high in the same year (Lawn *et al*., 2016; Kassebaum *et al*., 2016), despite medical assistance.

#### (d) Female mortality

**Fig. 6:**
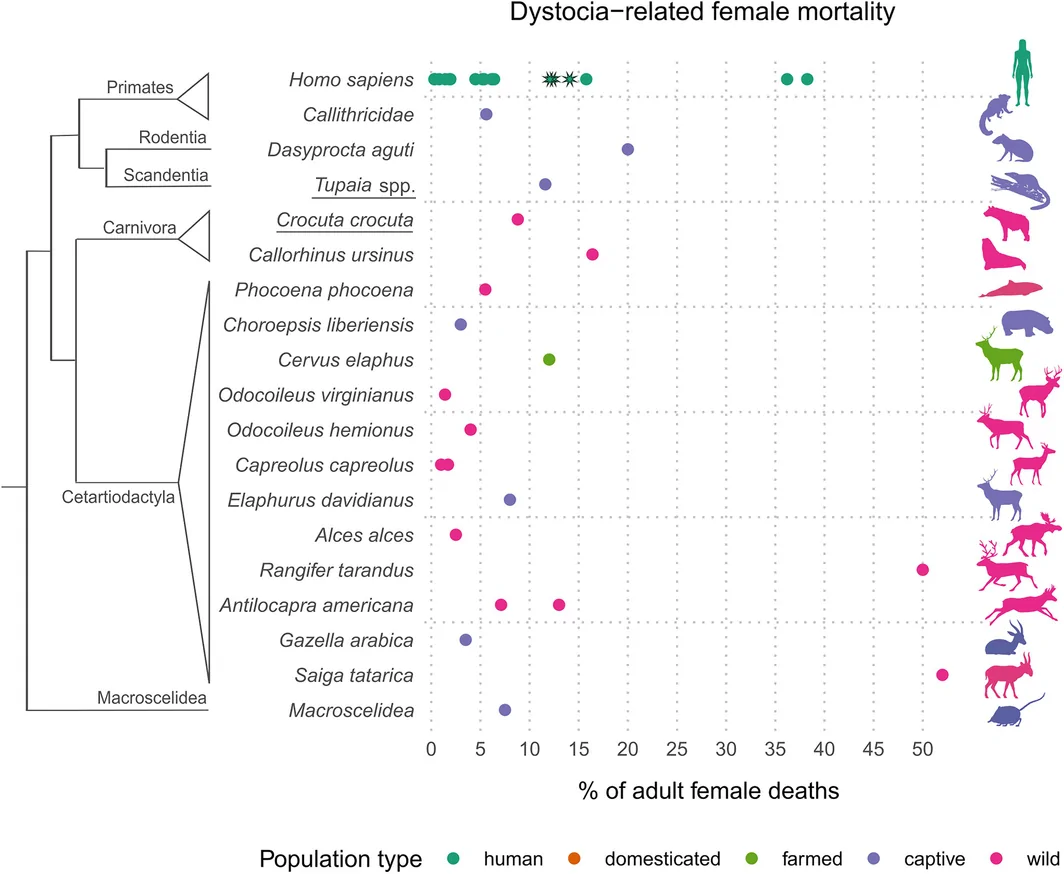
Proportion of adult female mortality attributed to dystocia across placental mammals. Altricial taxa are underlined. Each dot represents a study, population, or study period. Adult female mortality is defined as all deaths of sexually mature females (non‐human mammals) or as all female deaths between 15 and 49 years of age (humans, dots). Stars represent three hunter–gatherer populations living in non‐medicalised settings. Human dots represent HIV‐unrelated maternal deaths attributable to dystocia and other pregnancy‐ and birth‐related causes as a proportion of adult female mortality. The values for captive agoutis (*Dasyprocta aguti*) and wild reindeer (*Rangifer tarandus*) derive from small samples and are likely not representative of the entire species. Female mortality of the saiga antelope (*Saiga tatarica*) is specific to the birth season and is likely not representative of year‐round mortality. Female mortality >35% in humans (*Homo sapiens*) derive from western and central sub‐Saharan Africa. Mammal orders are given in the cladogram (branches not scaled) and silhouettes are from PhyloPic.org (public domain) or by the author (not to scale).

That parturition is one of the most perilous events in the life of a female placental mammal is evidenced by high rates of dystocia‐related adult female mortality, including in wild mammal populations (Fig. 6). In a well‐sampled wild colony of fur seals (*Callorhinus ursinus*), dystocia – mostly due to obstructive causes – was very prevalent and found to be the second‐most common cause (16%) of adult female mortality during the breeding season, and the leading cause of perinatal mortality (Spraker & Lander, 2010). In the pronghorn (*Antilocapra americana*), 7–13% of adult female mortality was attributable to fetopelvic disproportion and fetal maldisposition (Jacques *et al*., 2007; Kauth, 2017). Nearly one out of ten female wild spotted hyena (*Crocuta crocuta*) are estimated to die during their first birth (Frank, Weldele & Glickman, 1995). Human hunter–gatherer populations, such as the Agta (Philippines), the Hiwi (Venezuela), and the Hadza (Tanzania), exhibit similarly high mortality rates (10–15%). These are higher than in most other human populations, likely because the former, non‐medicalised societies do not engage in obstetrical intervention (Early & Headland, 1998; Blurton Jones *et al*., 2002; Hill *et al*., 2007). The large variation in female mortality rates in humans, from low in high‐income settings to high in low‐income countries (Fig. 6), partly reflects disparities in access to emergency obstetric services (Global Burden of Disease Collaborative Network, 2022; World Health Organization, 2023). Together, these data underscore the significance of birth complications as a major mortality pressure for female mammals, including humans, in non‐medicalised settings.

#### (e) Fetopelvic disproportion

**Fig. 7:**
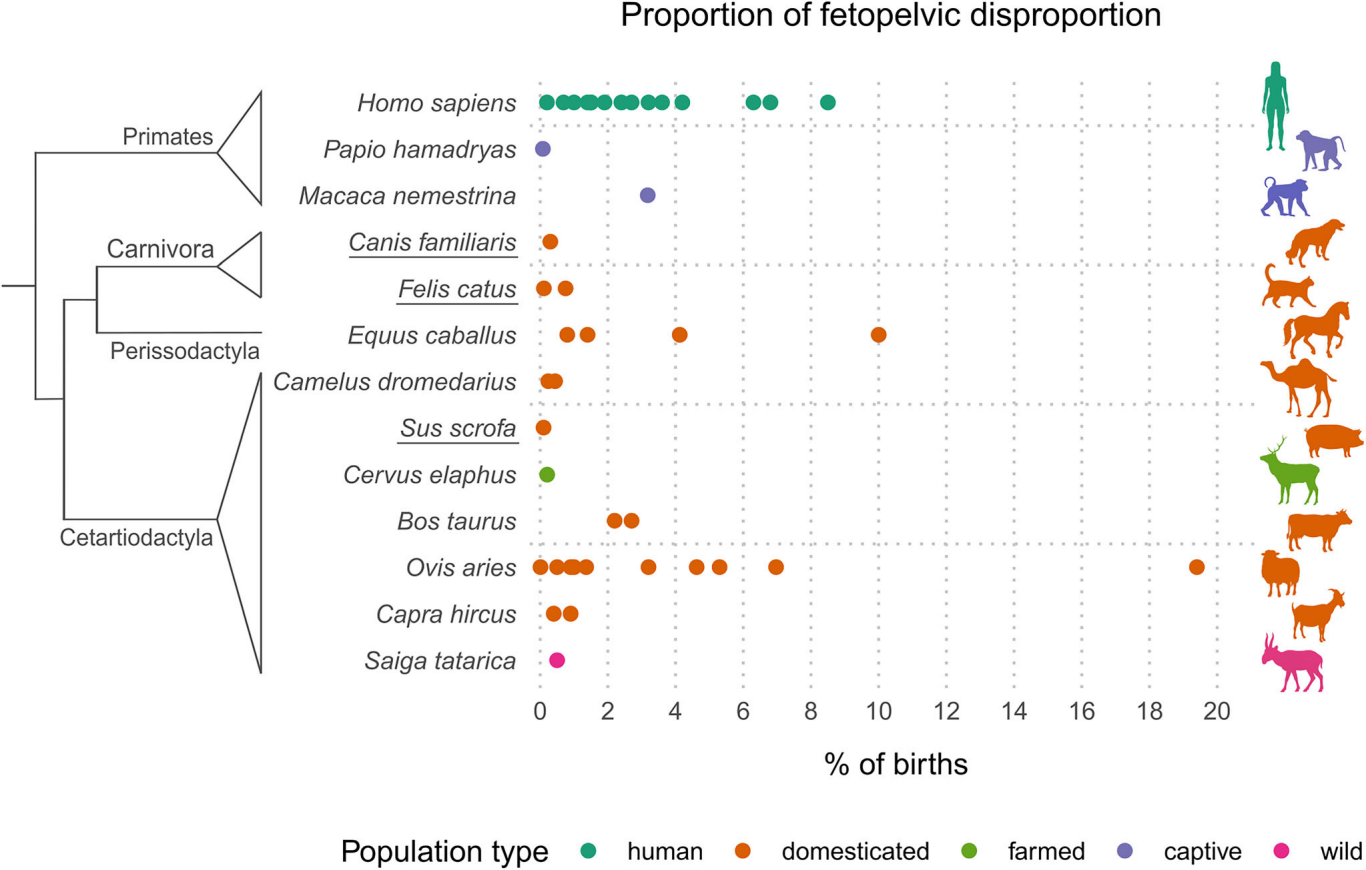
Proportion of fetopelvic disproportion among all births across placentals. Altricial taxa are underlined. Each dot represents a study, population, or study period. Most data for humans (*Homo sapiens*) derive from hospital populations, which are not necessarily representative of the general population. Some were also based on obstructed and/or prolonged labour rather than confirmed fetopelvic disproportion. Values ≥10% for horses (*Equus caballus*) and sheep (*Ovis aries*) reflect specific breeds or biased samples (e.g. from a veterinary clinic) and are unlikely to be representative of the entire species. The incidence of fetopelvic disproportion (0.09%) in the hamadryas baboon (*Papio hamadryas*) is likely an underestimate. Mammal orders are given in the cladogram (branches not scaled) and silhouettes are from PhyloPic.org (public domain) or by the author (not to scale).

Fetopelvic disproportion occurs predominantly in species with precocious offspring born at an advanced developmental stage, such as humans, domestic livestock, and non‐domesticated ungulates, such as deer (Fig. 7, Data S1). Disproportion is common in commercial, especially primiparous, livestock likely as a result of selective breeding for large offspring size and early breeding of females (Mee, 2008; Dwyer & Bünger, 2012; Tsaousioti *et al*., 2023). Humans display values encompassing the total range of mammalian variation. Some human data may overestimate true disproportion when diagnosed on the basis of obstructed or even simply prolonged labour (Dumont *et al*., 2001; Toh‐adam, Srisupundit & Tongsong, 2012) (see Section II.2). Nonetheless, high rates of fetopelvic disproportion are known from sub‐Saharan and South Asian communities with poor nutritional and health conditions, early age of marriage and first reproduction, and pelvic deformities, all of which are risk factors for feto‐maternal mismatch (Tolentino *et al*., 2019; Wells *et al*., 2021; Anikwe *et al*., 2022). While not unusual in low‐income settings today, these conditions may not be representative of human childbirth across time and space.

Fetal maldisposition may be secondary to fetal oversize. For instance, larger‐than‐average lambs (*Ovis aries*) are likely to be maldisposed with one or more forelimbs retracted, apparently due to insufficient intrauterine space, whereas smaller‐than‐average lambs are more likely to show other forms of maldisposition (Dwyer, 2003). An association between high birth weight and fetal malposture is also known in goats (Ismail, 2017) and is consistent with descriptions of dystocic births in wildebeest (*Connochaetes gnou*) (Amand *et al*., 1971), two deer species (Audigé *et al*., 2001a; Su *et al*., 2010), and giraffe (*Giraffa camelopardalis*) (Snyder & Richard, 1997). The incidence of fetus‐based fetopelvic disproportion may thus be underestimated in non‐human mammals.

#### (f) Dystocia in the wild

First‐hand observations of dystocic births exist for at least six species of wild primates, mostly Old World monkeys (Newton, 1990; Turner *et al*., 2010; Pandey *et al*., 2016; Daneze *et al*., 2016; Nguyen *et al*., 2017; Sivan *et al*., 2022; Pillay & Downs, 2024; Roura‐Torres *et al*., 2024). In most cases, the stage of fetal expulsion was significantly prolonged and accompanied by frequent straining by the female. In a primiparous mandrill (*Mandrillus sphinx*) and a Japanese macaque (*Macaca fuscata*) of unknown parity there were clear signs of fetopelvic disproportion due to the fetal head (Turner *et al*., 2010; Roura‐Torres *et al*., 2024). All these dystocic births were associated with both maternal and neonatal mortality or morbidity.

Taken together, cases of severe dystocia, i.e. leading to morbidity or mortality, have been observed in wild populations of over 30 non‐primate mammal species spanning diverse clades, including elk (*Cervus canadensis*) (Lehman *et al*., 2012), giraffe (Kaitho *et al*., 2011), three‐toed sloths (*Bradypus variegatus*) (da Silva & Rocha, 2015), southern right whales (*Eubalaena australis*) (Sironi *et al*., 2019), and African elephants (*Loxodonta africana*) (Moss, 2001) (see also Figs 3, 4, 5, 6, 7). Primiparous, but not multiparous, spotted hyenas experienced severe dystocia in 36% of captive births, with veterinary intervention preventing maternal mortality in half of those cases (Frank & Glickman, 1994). In the wild, female hyena mortality is highest in the year of first reproduction due to the risk of complications during a female's first birth (Frank *et al*., 1995). This implies that dystocia is an important cause of death in this species. Female spotted hyenas possess a clitoris resembling a penis, which – together with behavioural aggressiveness – plays a pivotal role in dominance hierarchies and associated feeding and breeding benefits. However, relatively large pups have to be born through this clitoris, which is not well aligned with the outlet of the bony pelvis (Frank *et al*., 1995). The fitness benefits of the highly derived genital morphology of female spotted hyenas thus come with a considerable risk of parturition‐related mortality and fitness costs.

#### (g) Relationship with brain size

The best metric to express obstetric challenge due to brain size across placentals remains unclear. Species vary extensively in adult brain and body size, degree of brain development at birth, and skull shape. Expressing neonatal brain size as a fraction of adult brain size can be misleading: human neonates reach ‘only’ 20–30% of adult brain size at birth (Barton & Capellini, 2011; DeSilva *et al*., 2017), yet cephalopelvic disproportion is common. By contrast, other mammals in the current sample achieve 12–72% of adult brain size at birth (Barton & Capellini, 2011). Similarly, neonatal brain mass as a proportion of female body mass ranges from 0.04 to 0.20% in precocial livestock, compared to 0.5% in humans (Capellini, Venditti & Barton, 2011), yet all experience frequent feto‐maternal mismatch (Fig. 7). These patterns indicate that obstructive dystocia, including fetopelvic disproportion, occurs across a wide range of brain sizes. Factors such as relative size or body shape of the neonate likely contribute substantially to birth difficulty.

### (3) Underlying causes and risk factors for dystocia

There are many similarities in the underlying causes and risk factors for dystocia across mammalian taxa, including humans. Available evidence indicates that dystocia is more commonly due to obstruction of the fetus than due to the lack of initiation or progress of uterine contractions (Table 2). In turn, obstructive dystocia is mainly due to fetal maldisposition, probably because of the many ways in which the fetal head and limbs can misalign with the birth canal. Fetopelvic disproportion is also an important contributor in some species (Fig. 7, Table 2, Data S1), arising from the mismatch between fetal and maternal dimensions.

**Table 2:** Examples of underlying causes of dystocia shared among humans and other placental mammals.

	Taxa^a^	Sources
Obstructive dystocia more common than functional dystocia	**Humans (multiparae)**, cattle, sheep, goats, horses, dromedary, some dog breeds, guinea pigs, pigtailed macaques, vervet monkeys, red deer, giraffe. Likely also ferrets, laboratory mice, spotted hyena, wild saiga antelope, baboons, chimpanzees, and other primates.	Smythe (1986); Darvelid & Linde‐Forsberg (1994); Frank & Glickman (1994); Ginther & Williams (1996); Snyder & Richard (1997); Harkness *et al*. (2002); Jackson (2004); Mahoney (2005); Zhu *et al*. (2006); Mee (2008); Fowler (2010); Kavanagh *et al*. (2011); Stockinger *et al*. (2011); Mayer *et al*. (2015); Barros (2016); Ali *et al*. (2016); Jacobson *et al*. (2020); Tsaousioti *et al*. (2023); Cheleuitte‐Nieves *et al*. (2025)
Majority of obstructive dystocia due to fetal^b^, not maternal^c^, factors	Cattle, sheep, goats, horses, pigs, llamas and alpacas, dromedary, rabbits, pigtailed macaques, baboons, vervet monkeys, giraffe, mink, wild saiga antelope, and capybara. **Unclear in humans**. Maternal pelvis plays an equally or more important role in dogs, guinea pigs, squirrel monkeys, and possibly in elephants and African pygmy hedgehogs. Soft tissue of maternal reproductive tract is important in wild spotted hyena.	As above, plus Aksel & Abee (1983); Schneider & Hunter (1993); Frank & Glickman (1994); Cueto (1999); Connolly *et al*. (2003); Hildebrandt *et al*. (2006); Münnich & Küchenmeister (2009); Fowler (2010); Tsvieli *et al*. (2012); Gleeson *et al*. (2019)
Functional dystocia more common (notably uterine inertia)	**Humans (primiparae)**, domestic pigs, some dog breeds, and cats.	Jackson (2004); Zhu *et al*. (2006); Noakes *et al*. (2009); Münnich & Küchenmeister (2009)

^a^ Non‐human mammal data come from domesticated, farmed, or captive animals, unless specified otherwise. Taxa typically considered to be altricial are underlined (here, this includes pigs but not humans).

^b^ Most commonly fetal maldisposition in non‐human mammals, although fetal oversize also important.

^c^ For example, a small and/or immature maternal bony pelvis.

#### (a) Primiparity

Several factors predispose to dystocia in humans and other mammals (Table 3). Across humans and other placental mammals, giving birth for the first time (primiparity) is a key maternal risk factor. However, the underlying mechanisms are complex. Primiparity, young age and small maternal size often interact in their effect on dystocia risk (Jacobson *et al*., 2020; Wells *et al*., 2021). In domestic ruminants and camelids, a young age at first reproduction increases the likelihood of fetopelvic disproportion, which is typically attributed to maternal pelvic immaturity and a small birth canal (Mee, 2008; Su *et al*., 2010; Purohit, 2012; Jacobson *et al*., 2020). The interaction of primiparity with young age in humans is less clear. Adolescent, pre‐adult primiparae tend to deliver smaller fetuses, experience faster labour, and have lower rates of obstructed labour and caesarean delivery (Scholl, Hediger & Belsky, 1994; Kawakita *et al*., 2016; Siakwa, Nyarko‐Sampson & Bruce, 2020).

**Table 3:** Examples of risk factors shared among humans and other placental mammals.

Dystocia risk factor	Taxa^a^	Sources
Primiparity	**Humans**, cattle, sheep, goats, deer, llamas and alpacas, dromedary, pigs, horses, rabbits, laboratory mice, tenrec, elephants, squirrel monkey, common marmoset, long‐tailed macaques, wild spotted hyena, possibly sifaka, as well as dogs, and guinea pigs and elephants in case of old primiparae.	Poole & Evans (1982); Glickman *et al*. (1993); Stoller (1996); Harkness *et al*. (2002); Jackson (2004); Haigh *et al*. (2005); Woodbury *et al*. (2006); Zhu *et al*. (2006); Mee (2008); Noakes *et al*. (2009); Münnich & Küchenmeister (2009); Hashim *et al*. (2012); Purohit (2012); Sabbagh *et al*. (2014); Offerhaus (2015); Levallois & De Marigny (2015); Hartley & Stanley (2016); Cassady *et al*. (2018); Jacobson *et al*. (2020); Houck & Harrison (2021); Cheleuitte‐Nieves *et al*. (2025)
Small maternal size/stature^b^	**Humans**, small dog breeds, horse, rabbits, and possibly pigs.	Tsu (1992); Ginther & Williams (1996); Rozenholc *et al*. (2007); Noakes *et al*. (2009); Münnich & Küchenmeister (2009); Toh‐adam *et al*. (2012); O'Neill *et al*. (2017); Gleeson *et al*. (2019)
Low and high birth weight	**Possibly humans**, pigtailed macaques, cattle, sheep, pigs, and possibly cats, mink, tenrecs, and wild saiga antelope.	Godfrey & Oliver (1978); Schneider & Hunter (1993); Jackson (2004); Noakes *et al*. (2009); Stockinger *et al*. (2011); Dwyer & Bünger (2012); Barros (2016); Noli *et al*. (2019); Jacobson *et al*. (2020); Černá *et al*. (2024)
High or excess energy intake during pregnancy	**Humans**, squirrel monkeys, vervet monkeys (including wild vervets in urban environments), marmosets and tamarins, guinea pigs, capybara, cattle, and elk.	Maree (1986); Friedel & Hudson (1994); Debyser (1995); Jackson (2004); Alvarez & Kravetz (2006); Moses *et al*. (2006); Masters (2010); Kavanagh *et al*. (2011); Pillay & Downs (2024)
Maternal overweight	**Humans**, callithrichid primates, possibly baboons, cattle, sheep, red deer, elk, guinea pigs, likely also degus, and elephants.	Friedel & Hudson (1994); Audigé *et al*. (2001a); Mee (2008); Hermes *et al*. (2008); Schlabritz‐Loutsevitch *et al*. (2008); Johnson‐Delaney (2010); Masters (2010); Wells (2017); Wells *et al*. (2018); Jacobson *et al*. (2020); Muhammad *et al*. (2022)

^a^ Non‐human mammal data come from domesticated, farmed, or captive animals, unless specified otherwise. Species typically considered to be altricial are underlined (here, this includes pigs but not humans).

^b^ Specifically as a risk factor for obstructive dystocia, typically fetopelvic disproportion.

However, these human patterns are context dependent. Teenage girls in low‐income settings are more likely to have a contracted birth canal associated with malnutrition while also being less likely to receive antenatal care, both of which are linked to higher rates of fetopelvic disproportion, protracted labour, and caesarean delivery (Rossiter *et al*., 1985; Scholl *et al*., 1994; Adu‐Bonsaffoh, Tunçalp & Castro, 2022; Muhammad *et al*., 2022; Emagneneh *et al*., 2025). It has also been suggested that the lower gynaecological age – a shorter period between menarche and first birth – of adolescent girls in low‐income compared to high‐income countries is associated with greater pelvic immaturity and contributes to higher rates of obstructed labour (World Health Organization, 2004).

Primiparity remains a risk factor irrespective of age. In adult women and cattle, for example, both functional and obstructive dystocia tend to occur more frequently in first compared to subsequent births (Zhu *et al*., 2006; Noakes *et al*., 2009; Laughon *et al*., 2014; Anikwe *et al*., 2022). In some taxa, primiparity elevates the risk of dystocia especially when associated with advanced maternal age (Table 3). For example, old primiparous guinea pigs and elephants have an increased risk of obstructive dystocia due to fusion and reduced flexibility of the pubic symphysis, a condition associated with delayed first reproduction (Hildebrandt *et al*., 2006; DeCubellis, 2016).

#### (b) Maternal size

Within species, smaller maternal body size is generally associated with larger relative neonatal size. Obstructive dystocia, especially fetopelvic disproportion, is more likely in small breeds of domestic animals (e.g. in dogs and horses *Equus caballus*) and in women of small stature (<145 to <155 cm depending on the population), regardless of age (Table 3). In humans, short maternal stature and small pelvic dimensions can be the result of poor childhood nutrition and other adverse conditions in early life that stunt skeletal growth and increase the risk of obstructed labour later in life (Wells, 2017). The same mechanism of ecological stress likely influences variation in obstructive dystocia prevalence in other mammals. In wild sika deer (*Cervus nippon*), multiple females with smaller‐than‐average body dimensions died of obstructive dystocia with normal‐sized fetuses impacted in the birth canal. The majority were primiparae. The authors attributed these incidences of obstructive dystocia to the females' stunted growth caused by adverse climatic conditions and nutritional stress a few years earlier (Takahashi *et al*., 2005).

Similar patterns are also evident *between* species, suggesting that adult body size influences dystocia risk not only within populations but also across taxa through allometric scaling. Across both precocial and altricial placental mammals, smaller‐bodied placentals tend to have larger neonates relative to maternal body mass (Grunstra *et al*., 2019). Among altricial rodents, for example, dystocia is well‐known in small‐bodied laboratory mice whereas in larger‐bodied laboratory rats it is rare (Kohn & Clifford, 2002; Cheleuitte‐Nieves *et al*., 2025). This difference in birth difficulty is further supported by the presence of a flexible and ‘open’ pubic symphysis in female mice – an obstetric advantage that likely evolved to enlarge the fetopelvic fit – that is absent in female rats (Ortega *et al*., 2003).

#### (c) Fetal size

On the fetal side, high birth weight (e.g. fetal macrosomia) is a principal risk factor for fetopelvic disproportion across placentals. Mechanistically, both the size of the maternal birth canal and fetal size are relevant for causing disproportion. In the literature it is more often attributed to an oversized fetus than a small maternal pelvis (Kock *et al*., 1988; Darvelid & Linde‐Forsberg, 1994; Donnelly & Quimby, 2002; Zaborski *et al*., 2009; Hartley & Stanley, 2016; Gleeson, Sanchez‐Migallon Guzman & Paul‐Murphy, 2019; Gupta *et al*., 2020). Nevertheless, variation in maternal birth canal shape between individuals or breeds is associated with the prevalence of dystocia in humans, cattle, sheep, cats and dogs (McSporran & Fielden, 1979; Noakes *et al*., 2009; Zaborski *et al*., 2009; Dwyer & Bünger, 2012; Starrach *et al*., 2023; Černá *et al*., 2024).

Surprisingly, low birth weight (e.g. a small‐for‐gestational‐age fetus) has also been found to contribute to dystocia in humans and other mammals (Table 3). Small fetuses can increase the risk of fetal maldisposition and may also play a role in uterine inertia (Luterkort *et al*., 1986; Jackson, 2004; Linde Forsberg & Persson, 2007; Ström Holst & Frössling, 2009; Dwyer & Bünger, 2012; Barros, 2016; Jacobson *et al*., 2020; Černá *et al*., 2024).

#### (d) Nutrition

A maternal environment of nutritional excess and high‐calorie foods either before or during pregnancy can result in fetal overgrowth, or fetal macrosomia (Table 3). Humans, other primates, and rodents are sensitive to a diet rich in carbohydrates, especially sugars, which may cause gestational diabetes and overdeveloped fetuses. The effects of maternal nutrition on fetal development are complex, however. For example, a high body mass index (BMI), a proxy for adiposity, is associated with an increased risk of fetal overgrowth and fetopelvic disproportion (Cedergren, 2009; Gaudet *et al*., 2014; Wells, 2017). Yet even in a Nepalese population where most women were underweight or within the ‘normal’ BMI range, obstructed labour risk increased with BMI (Wells *et al*., 2021).

Human studies also link maternal hyperglycemia and gestational diabetes to pre‐term birth, an extreme example of shortened gestation (reviewed in Wells *et al*., 2024). Similarly, one study on farmed red deer showed that pregnant females with unrestricted access to food shortened their gestation by an average of 8 days compared to those on restricted diets, with no recorded cases of dystocia (Asher *et al*., 2005). These findings suggest the existence of physiological mechanisms that shorten gestation in energy‐rich maternal environments, possibly to reduce the risk of fetopelvic disproportion (Wells *et al*., 2024). However, if such mechanisms exist, they can be overwhelmed or may play only a minor role in controlling fetal growth in some species, as evidenced by high rates of fetal macrosomia and fetopelvic disproportion in at‐term fetuses recorded in humans, captive primates, and domesticated rodents (Harkness, Murray & Wagner, 2002; Masters, 2010; Jekl, Hauptman & Knotek, 2011; Kavanagh *et al*., 2011; Wells *et al*., 2024).

#### (e) Other risk factors

There are additional shared risk factors for obstructive dystocia. For example, small litters are associated with larger individual fetuses and fetopelvic disproportion in several litter‐bearing species (Donnelly & Quimby, 2002; Noakes *et al*., 2009; Ström Holst & Frössling, 2009; Gleeson *et al*., 2019), while large litters increase the risk of fetal maldisposition (Jackson, 2004; Noakes *et al*., 2009; Dwyer & Bünger, 2012). A similar pattern is observed in human singleton *versus* twin births (Obiechina *et al*., 2011). Male fetuses, being larger on average, also increase the risk of obstruction in both humans (Muleta, Rasmussen & Kiserud, 2010; Patumanond, Tawichasri & Khunpradit, 2010; Wells *et al*., 2021) and several livestock species (Noakes *et al*., 2009).

## IV. DISCUSSION

Human childbirth carries risks of complications and mortality. However, birth complications (dystocia) are not unique to humans but are widespread among placental mammals, irrespective of phylogeny and mode of locomotion. Prevalence and mortality rates related to birth complications in humans either aligned with or overlapped with ranges reported for other taxa. Dystocia, including fetopelvic disproportion, even occurs at meaningful frequencies in wild populations, demonstrating that, contrary to assumptions (Roy, 2003; Mee, 2008), natural selection has not eliminated this risk (see also Laudicina *et al*., 2025). Evidence from strictly aquatic mammals lacking a true bony pelvis underscores the importance of soft tissue anatomy (e.g. pelvic musculature) and shows that obstructive dystocia is not solely contingent on a bony birth canal. Underlying causes and risk factors – such as maternal and fetal characteristics as well as nutritional status – are also very similar in humans and other mammals. This highlights a shared physiology of pregnancy, fetal growth, and birth among placentals.

Together, these findings challenge the long‐held assumption that difficult birth is an evolutionary anomaly or a uniquely human problem, complementing recent comparative work (Grunstra, 2024; Laudicina *et al*., 2025). Rather, the data suggest that birth in many placental mammals is characterised by a persistent baseline frequency of complications despite the obvious fitness costs. The outcome of dystocia is best understood not as a discrete evolutionary trait, but as an emergent reproductive complication that arises when anatomical, physiological, and environmental factors combine to impede successful birth.

### (1) Life‐history trade‐offs: the benefits and costs of large offspring

This comparative perspective reframes the evolutionary question from ‘why is human birth so difficult?’ to ‘why has natural selection not eliminated birth complications in mammals generally?’. More than a burden of human evolution, the ubiquity of obstructive dystocia calls for an expanded perspective of mammalian life‐history trade‐offs.

Fitness trade‐offs in mammalian reproductive investment are a major theme in life‐history theory, reflecting the fundamental constraint of limited resources that forces allocation decisions (Stearns, 1992). The well‐known fast–slow life‐history continuum encompasses trade‐offs between (*i*) reproductive rate and reproductive lifespan, and (*ii*) litter size and birth size (also known as the ‘quantity–quality’ trade‐off). Small and altricial mammals typically trade off the benefits of fast reproduction and large litters against a short lifespan and high mortality rates, whereas precocial and larger‐bodied mammals tend to invest in small litters of well‐developed offspring with lower juvenile mortality at the expense of a slower reproductive rate (Promislow & Harvey, 1990; Stearns, 1992). These and many other classic life‐history trade‐offs are rooted in energetic constraints on growth, reproduction, and survival.

Based on the present work, I propose the existence of another key fitness trade‐off in mammalian life history, but one rooted in the capacity of the maternal pelvis. The mechanical constraint imposed by the birth canal forces a compromise between postnatal survival benefits of large offspring and intrapartum mortality risk. As selection for large birth size increases, so does the risk of obstructive dystocia and associated mortality and morbidity – of both the female and the offspring. Many placental mammals likely face this fitness trade‐off, yet it is rarely considered in mammalian life‐history evolution (but see Laudicina *et al*., 2025). How females optimise offspring size must balance a complex combination of variables that includes not only growth rates, litter size, ecological conditions, and energy availability (Hayssen & Orr, 2017), but also mechanical constraints and the mortality risks of parturition.

The prevalence of obstructive dystocia, including fetopelvic disproportion, was found to be highest in species with precocial offspring. In many mammals, including humans, larger neonates have improved survival prospects, better thermoregulation, and greater competitive ability (e.g. Barker *et al*., 1989; Sibly & Brown, 2009; Calambokidis & Gentry, 1985; Hodge *et al*., 2009). These advantages increase maternal (inclusive) fitness and therefore create selective pressure for increased offspring size. In all mammals, pelvic morphology is ultimately constrained, although the strength and nature of these constraints likely vary depending on species‐specific positional behaviour, weight‐bearing demands, and other biomechanical factors. The maternal pelvic canal therefore cannot expand indefinitely to accommodate the fetus without compromising other important functions. The resulting evolutionary tension likely maintains offspring size near the maximum that fits through the average maternal birth canal, which comes at the inevitable cost of occasional feto‐maternal mismatch and other obstructive complications.

The evolutionary trade‐off between neonatal size and pelvic constraints exemplifies cliff‐edge selection, previously proposed to explain obstructed labour in humans (Mitteroecker *et al*., 2016), and implies that such dynamics may be widespread among placental mammals. While most evident in precocial mammals, altricial placentals may experience the same trade‐off. In altricial species, higher birth weight also decreases the probability of postnatal mortality and directly or indirectly reduces the chances of fetal maldisposition (Godfrey & Oliver, 1978; Jackson, 2004). Observations of captive mice, treeshrews, and pigs show that the risk of fetopelvic disproportion also exists in altricial and litter‐bearing species (Jackson, 2004; Clancy *et al*., 2013; Cheleuitte‐Nieves *et al*., 2025).

**Fig. 8:**
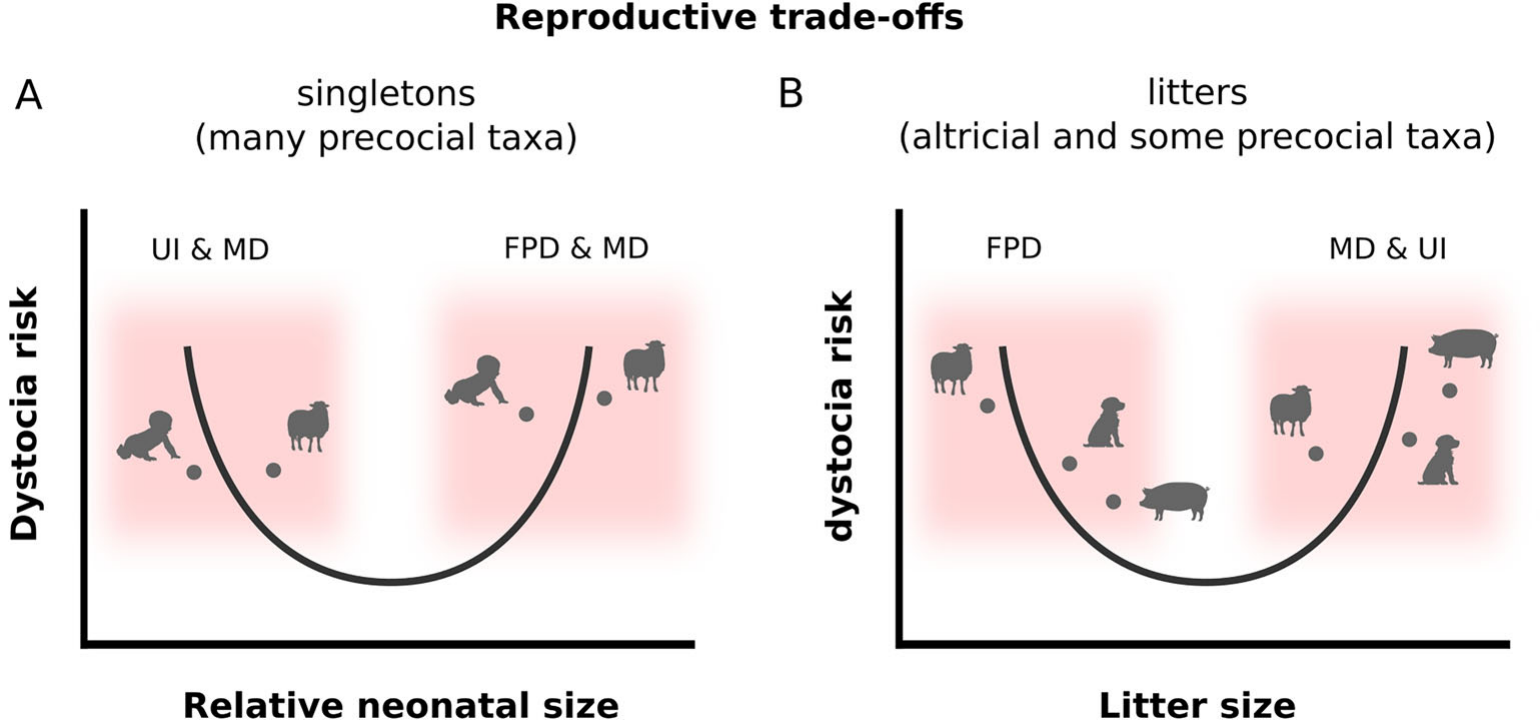
Schematic illustration of two proposed fitness trade‐offs in placental mammals between dystocia risk and (A) newborn size relative to maternal size for singleton births and (B) litter size. Within species, dystocia risk is hypothesised to increase progressively towards both high and low values of relative neonatal size and litter size. Both traits serve as rough proxies for maternal fitness: larger neonatal size is generally linked to greater postnatal offspring survival, while litter size reflects a trade‐off between offspring quantity and quality. Depending on ecological conditions, smaller or larger litter size maximises the number of successful offspring. As these traits vary, the underlying causes of dystocia are expected to differ, including uterine inertia (UI), fetal maldisposition (MD), and fetopelvic disproportion (FPD). The approximate positions of a few example taxa are included. Sheep appear in both panels because it is relatively well understood how dystocia risk and type vary with lamb size and litter size. Silhouettes from PhyloPic.org (public domain).

In precocial species bearing singletons, small neonatal size may also elevate dystocia risk, through fetal maldisposition and possibly uterine inertia, rather than fetopelvic disproportion. Small fetal size and, hence, small uterine volume may offer insufficient mechanical stimulation to the uterine wall, inhibiting contractions (Linde Forsberg & Persson, 2007; Noakes *et al*., 2009; Ström Holst & Frössling, 2009). Evidence from sheep suggests that both large and small fetal size can cause maldisposition at birth (Dwyer, 2003; Dwyer & Bünger, 2012; Jacobson *et al*., 2020). Thus, in singleton‐bearing species, dystocia risk appears to increase at both ends of the fetal size spectrum, creating a U‐shaped relationship between relative neonatal size and risk of intrapartum complications (Fig. 8A).

The present review has also elucidated a similar but more complex fitness trade‐off in litter‐bearing placentals. In litter‐bearing species, which are often altricial but sometimes precocial (e.g. guinea pigs, some deer, and saiga antelope), the trade‐off centres on litter size rather than individual neonatal size. In many taxa, smaller litters comprise larger individual fetuses, which may enhance neonatal viability but increases the risk of fetopelvic disproportion and intrapartum mortality (Darvelid & Linde‐Forsberg, 1994; Noakes *et al*., 2009; Münnich & Küchenmeister, 2009). Conversely, larger litters can confer fitness benefits by increasing the number of potential surviving and reproducing offspring (Lack, 1947; Stearns, 1992), but they also increase the risk of both functional and obstructive dystocia. Uterine inertia may result from uterine overdistension or fatigue (Jackson, 2004; Noakes *et al*., 2009; Černá *et al*., 2024). Larger litters can also cause ‘crowding’ of the fetuses *in utero*, and lead to simultaneous presentation of multiple fetuses and other forms of fetal maldisposition (Dwyer, 2003; Jackson, 2004). Thus, both small and large litters are associated with elevated intrapartum mortality risk (Fig. 8B).

Anatomical variation among lineages may influence how these trade‐offs are expressed. For example, cetaceans and female bats possess reduced bony pelvic girdles. Consequently, their bony pelves impose a weaker mechanical constraint on their large offspring during birth (Crelin, 1969). They are therefore expected to show a smaller increase in the risk of fetopelvic disproportion with increasing neonatal size. However, obstructive dystocia is known to occur in both lineages (Fig. 2), highlighting the importance of constraints imposed by soft tissue anatomy.

What do these patterns imply about dystocia risk in deep evolutionary history? It is possible that early placentals were already vulnerable to dystocia if they faced comparable life‐history trade‐offs involving offspring size and/or litter size, because their anatomy, physiology and development likely permitted multiple proximate pathways to occasional birth complications. Dystocia is also known in oviparous birds and reptiles (‘egg‐binding’), often due to an oversized or malformed egg or inadequate contractions (Kilham, 1972; Cuadrado *et al*., 2002; Bowles, 2002). This supports the notion that parturition difficulties in a broad sense may be an ancient amniote challenge (see also Fischer *et al*., 2021; Laudicina *et al*., 2025). Further research, including phylogenetic comparative methods, is needed to estimate the phylogenetic origin and magnitude of dystocia risk in ancestral placental mammals.

### (2) Plasticity and environmental modulation of dystocia risk

In addition to evolved trade‐offs, plastic responses to the environment introduce another layer of complexity. The ‘baseline’ risk or vulnerability to birth complications in humans and other mammals deriving from evolved traits interacts with contemporary ecological factors such as nutrition, disease load, and climatic stressors (Wells *et al*., 2012). Through their influence on maternal and fetal growth, such factors can exacerbate – or alleviate – a tight fetopelvic fit (Wells, 2015). Rapid ecological or nutritional transitions across generations that improve fetal growth relative to maternal development have been linked to global variation in c‐section rates – a proxy for obstructed labour (Zaffarini & Mitteroecker, 2019). Furthermore, malnutrition (both over‐ and undernutrition) can increase the risk of obstructive dystocia through multiple pathways. The ‘dual burden of malnutrition’, in which maternal stunting co‐occurs with maternal obesity and fetal macrosomia, is especially pronounced in low‐ and middle‐income countries, where caesarean deliveries are most frequent among women who are both short and obese (Wells *et al*., 2018, 2020). Macro‐ and micronutrient deficiencies also contribute to contracted birth canals in low‐resource settings, particularly across sub‐Saharan Africa, which poses a high risk for fetopelvic disproportion and maternal mortality (Teguete *et al*., 2012; Desta *et al*., 2022).

Plastic responses to local ecological conditions thus help explain the heterogeneity in dystocia and birth‐related mortality risks in humans (Wells, 2015, 2017) (see also Figs 3, 4, 5, 6, 7). Within‐species variation in dystocia metrics was also observed for domesticated mammals and captive wildlife (Figs. 3, 4, 5, 6, 7). Further comparative evidence showed that nutritional excess before and during pregnancy is a risk factor for fetal macrosomia and obstructive dystocia in several non‐human mammals (Table 3). These patterns suggest that even in non‐human mammals, local ecological conditions can modulate dystocia risk in ways that mirror human obstetric challenges.

Metabolic sensitivity to ecological signals enables females to tailor their own growth and reproductive investment to resource availability during their lifetime. This capacity was likely favoured in resource‐scare or unpredictable environments (Neel, 1962), enabling female mammals to modulate offspring size’ in response to local conditions. This developmental plasticity partly explains between‐population variation in human birth weight (Black *et al*., 2013) and inter‐seasonal variation in birth weight in seals (Arnbom, Fedak & Rothery, 1994). But the same sensitivity also increases the risk of fetopelvic disproportion under extreme nutritional conditions. The latter partially explains the higher rates of obstructed labour in high‐ and low‐income human contexts (Wells *et al*., 2018, 2021) and could be linked to periods of nutritional deprivation and dystocia in wild sika deer (Takahashi *et al*., 2005). Such cross‐species parallels suggest conserved physiological mechanisms linking pre‐ and postnatal growth trajectories to pelvic morphology and obstetric outcomes. Nevertheless, this topic remains underexplored and merits more detailed investigation.

### (3) Recontextualising human birth and the obstetrical dilemma

The ubiquity of dystocia among placental mammals dismantles the idea that human birth is uniquely challenging and undermines explanations that tie obstetric risk to traits unique to our lineage. This further implies that the evolutionary roots of difficult childbirth may lie deeper in our mammalian past than in our hominid ancestry, which calls for an expanded evolutionary perspective of human childbirth and the obstetrical dilemma.

Today, the term ‘obstetrical dilemma’ is used to refer to subtly different ideas. In a narrow sense (*sensu* Washburn, 1960), the obstetrical dilemma refers specifically to the challenge of fitting a large neonatal head through a pelvis adapted for bipedalism (Rosenberg, 1992; Haeusler *et al*., 2021). The latter has often been interpreted as an energetic constraint on pelvic breadth imposed by bipedal locomotion (e.g. Lovejoy, Heiple & Burstein, 1973; Rosenberg, 1992; Ruff, 1995; Dunsworth *et al*., 2012). More broadly, ‘obstetrical dilemma’ is used as a shorthand for a tight fetopelvic fit and/or difficult, risk‐prone childbirth, as well as to describe the evolutionary trade‐off in pelvic morphology that underlies childbirth difficulties (reviewed in Haeusler *et al*., 2021; Dunsworth, 2021). The data reviewed herein show that humans are not unique in having an obstetrical dilemma in a wider sense. Many other placental mammals have tight fetopelvic fits, whether due to a relatively large fetal head (e.g. monkeys, brachycephalic dogs and cats, and possibly seals), or due to the simultaneous presentation of the fetal head and forelimbs (e.g. ungulates). These cases illustrate that trade‐offs between offspring size (and shape) and the mechanical limits of the birth canal, as well as the associated risk of birth‐related mortality, are common among placental mammals. In this broader sense, many species face their own obstetrical dilemmas.

As shown here, fetopelvic disproportion does not depend on bipedalism, encephalisation, or a human‐like pelvic morphology. This calls into question the human‐centred view that energetic constraints on pelvic breadth imposed by bipedal locomotion are a prerequisite for obstetric risk, consistent with evidence that people with mediolaterally wider pelves are not at a metabolic disadvantage while walking (Wall‐Scheffler, 2012; Wall‐Scheffler & Myers, 2013, 2017; Warrener *et al*., 2015; Gruss, Gruss & Schmitt, 2017; Whitcome, Miller & Burns, 2017). At the same time, associations between wider and larger birth canals and pelvic floor dysfunction in women underscore that bipedal posture and other functional constraints remain relevant for pelvic morphology in humans (Sze *et al*., 1999; Handa *et al*., 2003; Brown *et al*., 2013; Stansfield *et al*., 2021a,b, 2025).

Humans owe much of their birth difficulties to a unique combination of a large neonatal brain and a relatively narrow and twisted birth canal. However, other mammals owe their birth difficulties to their own unique combination of pelvic and fetal factors. For example, most ungulates deliver large offspring with long and stiff limbs prone to malposition through a pelvis with limited joint flexibility (Grunstra *et al*., 2019). Spotted hyenas uniquely give birth to comparatively large pups through a long and winding birth canal culminating in a peniform clitoris, which usually ruptures during the first birth (Frank & Glickman, 1994; see also Dunsworth, 2016).

Furthermore, while humans are prone to cephalopelvic disproportion due to large neonatal brains, obstructive dystocia in many other mammals more often results from fetal maldisposition – although maldisposition can itself be associated with fetal oversize. In both cases, however, spontaneous resolution is rare, and maternal and/or fetal death is likely without intervention.

Another reason cited for the difficulty of human childbirth is that, beyond a tight fetopelvic fit, the human fetus typically must undergo multiple rotations in order to align with the complex geometry of the human birth canal (Rosenberg & Trevathan, 2002; Haeusler *et al*., 2021). This specific pattern of fetal rotation has not (yet) been observed in other mammals and is frequently taken to emphasise the uniqueness of human parturition. But mammalian pelvic morphology is diverse and other species may also have a pelvis that is especially challenging for parturition. For example, several species possess soft tissue structures in the reproductive tract that are known risk factors for obstructive dystocia, such as an occasional persistent hymen [e.g. in domestic pigs and elephants (Jackson, 2004; Kiso *et al*., 2017)], a vestibulovaginal or vaginal–vestibular sphincter [e.g. in llamas (*Lama glama*), alpacas (*L. pacos*), horses, and sheep (Rodriguez, Pearson & Tibary, 2014; Noakes *et al*., 2009), or a particularly tight and morphologically complex vagina in sirenians, which may help defend against forced copulation by males and/or prevent retrograde water flow (Hall *et al*., 2012; Orbach *et al*., 2018; Brennan, 2022). As such, the tendency to emphasise the morphological distinctiveness of human birth risks obscures distinct and important anatomical constraints in other species.

What sets humans apart is not the presence or even the frequency of dystocia, but the cultural responses it has elicited. Humans are the only species known to have developed systematic birth assistance, including midwifery and medical interventions such as c‐sections, to manage complications during labour (Trevathan, 2011). This biocultural adaptation must be acknowledged in comparative studies of parturition, because it highlights the exceptional degree to which humans were able to adapt socially and culturally to manage the risks inherent in their mode of reproduction.

Yet, a noteworthy parallel exists: routine obstetric management has become ingrained in animal husbandry and is often necessary in captive wildlife. Although these interventions are not cultural adaptations by non‐human taxa, they similarly reduce birth‐related mortality and relax natural selection on traits favouring eutocia (normal parturition). In this way, humans are reshaping not only their own reproductive trajectories but also those of other – especially domesticated – mammals.

## V. CONCLUSIONS

(1) Human childbirth can have adverse outcomes for mothers and their babies. Despite this reality, birth in humans is not uniquely difficult in a phylogenetic comparative context, contrary to popular belief. Difficult and risk‐prone parturition is a widespread evolutionary pattern among placental mammals, and humans are typical in terms of both the intrinsic risks associated with birth and the proximate factors that predispose to dystocia.
(2) Two newly identified life‐history trade‐offs underlie dystocia risk across mammals, with evolutionary compromises arising from both relative neonatal size and litter size. In precocial species, selection favours large, well‐developed offspring, but this increases the risk of fetopelvic disproportion and fetal maldisposition – a persistent, phylogenetically widespread outcome of a trade‐off between intrapartum and postnatal survival. In litter‐bearing species (often altricial mammals), dystocia risk is shaped by a trade‐off between the number and the size of the offspring: small litters with larger fetuses elevate the risk of fetopelvic disproportion, while large litters with smaller fetuses increase the risk of uterine inertia and fetal maldisposition.
(3) Cliff‐edge selection and phenotypic plasticity contribute to the persistence of dystocia across placental mammals. Natural selection cannot eliminate obstructive dystocia entirely, because cliff‐edge selection likely pushes neonatal size toward the obstetric maximum, leading to a small but consistent proportion of obstructed births. This risk is further amplified by the developmental plasticity elicited by environmental variability – both within individuals over the life course and between individuals or populations. Periods of nutritional transition and ecological mismatch are expected to be especially important risk factors for intrapartum complications in humans and placental mammals more broadly.
(4) Against a background of broadly similar susceptibility to birth complications, the degree to which those complications are realised varies along a continuum among placental mammals, shaped by anatomical, developmental, and ecological factors. Humans occupy one region of this spectrum, but not a uniquely distinct one. Variation in the frequency and causes of birth complications spans both interspecific and intraspecific levels, with substantial overlap between them – including in humans.
(5) Obstructive dystocia is not solely contingent on a bipedal pelvis – or even on a bony pelvis *per se*. These observations highlight the importance of soft tissue anatomy in mediating the risk of dystocia. This, in turn, suggests the existence of potential trade‐offs in female mammalian genital tract anatomy, such as pelvic floor structures, which warrants further study.

## Supporting information


**Data S1.** Supplementary data on dystocia in placental mammals.

## Data Availability

The data that supports the findings of this study are available in the supplementary material of this article.
